# 

**Published:** 1885-05

**Authors:** 


### BOOKS AND PAMPHLETS RECEIVED.

A TEXT-BOOK OF PATHOLOGICAL ANATOMY AND PATHOGENESIS. By Ernest Ziegler, Professor of Pathological Anatomy in the University of Tubingen. Translated by Donald MacAlister, M. A. M. B., member of the Royal College of Physicians, Fellow and Medical Lecturer of St. John's College, Cambridge. Part II. 1884: Winwood & Co., New York.

THE PHYSIOLOGICAL EFFECTS AND THERAPEUTICAL USES OF HYDRASTIS. By Roberts Bartholow, A. M., M. D., LL. D., Professor of Materia Medica and General Therapeutics in the Jefferson Medical College of Philadelphia; Author of "A Practical Treatise of Materia Medica and Therapeutics," of "A Treatise on the Practice of Medicine," of "A Manual of Hypodermic Medication," of "A Practical Treatise on the Application of Electricity to Medicine and Surgery," etc., etc. Reprint from *Drugs and Medicines of Virginia.*

DISEASES OF THE URINARY AND MALE SEXUAL ORGANS. By Wm. T. Belfield, M. D., Pathologist to the Cook County Hospital, Surgeon Genito Urinary Department Central Dispensary, Chicago; Physician to the Oakwood Retreat, Geneva, Wis.; Professor of Microscopy, Chicago College of Dental Surgery. 1884: Wm. Wood & Co., New York.

Report of Committee on School Hygiene in Tennessee. By Daniel F. Wright, M. D., of Clarksville, Tennessee, Member of State Board of Health, and Chairman of its Committee on the Subject. January 1, 1885.

The Therapeutics of the Respiratory Passages. By Prosser James, M. D., Lecturer on Materia Medica and Therapeutics at the London Hospital Medical College; Physician to the Hospital for Diseases of the Throat and Chest; late Physician to the North London Consumption Hospital; Consulting Physician to the Children's Home Infirmary; Corresponding Member of the Academies of Medicine of Lyons, Madrid and Barcelona, etc., etc. 1884: Wm. Wood & Co., New York.

Vital Statistics in Tennessee. A Report by J. D. Plunket, M. D., of Nashville, Tenn., Member of the State Board of Health and its Committee on Vital Statistics.

A Manual of the Medical Botany of North America. By Laurence Johnson, A. M., M. D., Lecturer on Medical Botany Medical Department University of the city of New York; Fellow of the New York Academy of Medicine, and of the New York Academy of Science; Member of the Committee on Revision of the Pharmacopœia of the United States; Member of the Torrey Botanical Club, etc. 1884: Wm. Wood & Co., New York.

Experimental Researches on Cicatrization in Blood Vessels after Ligature. By N. Senn, M. D., Milwaukee, Wisconsin. Reprint Transactions American Surgical Association. Vol. II. 1884.

Studies from the Pathological Laboratory of the University of Pennsylvania—No. X; The Pathogenesis of Secondary Tumors. By Henry Wile, A. B., M. D., Rochester, N. Y. Reprinted from the *Philadelphia Medical Times* for July 29, August 12 and 26, and September 9, 1882.

HUMAN OSTEOLOGY, Comprising a description of the Bones with Delineations of the attachments of the Muscles, the general and Microscopic Structure of Bone and its development. By Luther Holden, ex-President and Member of the Court of Examiners of the Royal College of Surgeons of England; Consulting Surgeon to St. Bartholomew's and the Foundling Hospital, assisted by James Shuter, F. R. C. S., M. A. M. B., Cantab.; Assistant Surgeon to the Royal Free Hospital; Late Demonstrator of Physiology, and Assistant Demonstrator of Anatomy at St. Bartholomew's Hospital, with numerous illustrations. Sixth Edition. 1885: Wm. Wood & Co., New York.

THIRD ANNUAL REPORT OF THE BOARD OF HEALTH of the city of Macon, Georgia. By J. Emmett Blackshear, M. D., Chairman.

KIRKE'S HAND-BOOK OF PHYSIOLOGY. By W. Morrant Baker, F. R. C. S. Surgeon to St. Bartholomew's Hospital and Consulting Surgeon to the Evelena Hospital for Sick Children; Lecturer on Physiology at St. Bartholomew's Hospital, and Late Member of the Royal College of Surgeons of England, and Vincent Donner Harris, M. D., Lond. Demonstrator of Physiology at St. Bartholomew's Hospital. Eleventh Edition, with nearly 500 illustrations. Volumes I–II. 1885: Wm. Wood & Co., New York.

SOME NOTES ON THE HISTOLOGY OF LUPUS VULGARIS. By Henry Wile, M. D. Reprinted from the Archives of Dermatology, Vol. VIII, No. 4, October, 1882.

THE VIENNA SCHOOL OF DERMATOLOGY. By Henry Wile, M. D. Reprint from *Philadelphia Medical Times.*

HAND-BOOK OF THE DISEASES OF THE SKIN. Edited by H. V. Ziemssen, M. D., Professor of Clinical Medicine in Munich; Editor of Ziemssen's Cyclopedia of the Practice of Medicine. Illustrated with eighty wood engravings and color prints. 1885: Wm. Wood & Co., New York.

MEDICAL JURISPRUDENCE IN DIVORCE. By Carl H. von Klein, A. M., M. D. Dayton, Ohio. Reprint from the Journal of the American Medical Association.

A PRACTICAL TREATISE ON DISEASE IN CHILDREN. By Eustace Smith, M. D., Fellow of the Royal College of Physicians; Physician to His Majesty the King of the Belgians; Physician of the East London Children's Hospital, and to the Victoria Park Hospital for Diseases of the Chest. 1884: Wm. Wood & Co., New York.

A CASE OF AINHUM. By Louis A. Duhring, M. D., Clinical Professor of Diseases of the Skin in the University of Pennsylvania; with Microscopic Examination, by Henry Wile, M. D.

A MANUAL OF OBSTETRICS. By Edward S. Partridge, M. D., Professor of Obstetrics, New York Past Graduate Medical School; Instructor in Obstetrics, College of Physicians and Surgeons, New York; Visiting Physician to the Maternity Hospital and Nursery and Child Hospital; Fellow of the New York Obstetrical Society, etc., with sixty illustrations. 1884: Wm. Wood & Co., New York.

ON THE PATHOLOGY OF PAGET'S DISEASE OF THE NIPPLE. By Louis A. Duhring, M. D., Professor of Diseases of the Skin in the University of Pennsylvania, and Henry Wile, M. D., Clinical Assistant in Dermatology in the University of Pennsylvania, and Assistant Physician to the Philadelphia Dispensary for Skin Diseases.

PHYSICIAN HIMSELF, and what he should add to his Scientific Acquirements in order to Secure Success. By D. W. Cathell, M. D., late Professor of Pathology, College of Physicians and Surgeons, Baltimore, Md. 1885: Cushings & Bailey, 262 W. Baltimore street, Baltimore, Md.

TYPHOID FEVER AND LOW WATER IN WELLS. By Henry B. Baker, M. D., Lansing, Mich. Reprinted from the Annual Report of the Michigan State Board of Health for the year 1884.

REMARKS ON TYPHOID FEVER IN THE YOUNG. By A. Jacobi, M. D., Clinical Professor of Diseases of Children in the College of Physicians and Surgeons in New York. Reprinted from the "Archives of Pediatrics."

INAUGURAL ADDRESS DELIVERED BEFORE THE NEW YORK ACADEMY OF MEDICINE. By A. Jacobi, M. D,. President of the Academy. Reprinted from *The Medical Record.*

MASSAGE; THE LATEST HANDMAID OF MEDICINE. By Benjamin Lee, A. M., M. D., Ph. D., Philadelphia. Extracted from the Transactions of the Medical Society of the State of Pennsylvania for 1884.

CLINICAL ILLUSTRATION OF THE VALUE OF COMBINING MOTION WITH EXTENSION IN THE TREATMENT OF DISEASE OF THE HIP-JOINT. By Benjamin Lee, A. M., M. D., Ph. D., of Philadelphia. Extracted from the Transactions of the Medical Society of the State of Pennsylvania for 1884.

ANNOUNCEMENT COOPER MEDICAL COLLEGE, San Francisco 1885, and ANNUAL REPORT OF MORSE DISPENSARY OF COOPER MEDICAL COLLEGE for 1884.

SEVENTH ANNUAL REPORT OF THE PRESBYTERIAN EYE, EAR AND THROAT CHARITY HOSPITAL, NO. 77 East Baltimore St., Baltimore.

ADDRESS IN MEDICINE, delivered before the Medical Society of the State of Pennsylvania, by W. H. Daly, M. D., at its Annual Meeting held in Philadelphia, May, 1884.

ELEVENTH ANNUAL REPORT OF THE SUPERINTENDENT OF THE CINCINNATI SANITARIUM, FOR THE YEAR ENDING NOVEMBER 30TH.

CASES OF DISEASE OF THE FRONTAL SINUS. By Charles J. Kipp, M. D., Newark, N. J.

TRANSACTIONS OF THE AMERICAN DERMATOLOGICAL ASSOCIATION, Eighth Annual Meeting, held at Highland Falls, near West Point, New York, on the 27th, 28th and 29th of August, 1884. Official Report of the Proceedings by the Secretary, W. T. Alexander, A. M., M. D.

Every physician who wants to keep posted on medical topics in the South, should subscribe for THE ATLANTA MEDICAL AND SURGICAL JOURNAL, *the journal of the Medical Association of Georgia*, and the leading journal in the South. Subscription price, $2.50 a year.

### PUBLISHERS' ANNOUNCEMENT.

The publishers of THE ATLANTA MEDICAL AND SURGICAL JOURNAL take very great pleasure in informing the friends of THE JOURNAL and the Profession generally, that they have made arrangements whereby THE JOURNAL becomes *The Journal of the Medical Association of Georgia.* Under this arrangement the papers of the Association, which have heretofore been published in book-form, will now be published monthly in this JOURNAL.

We will be able to furnish reprints of any papers, charging only for making the forms, press-work, paper and binding. We can furnish reprints at the following prices:

For eight pages, without cover, 100 copies, . . . . $ 3.30
Cover, $1.75 extra.
500 copies without cover, . . . . . . . . . . . . . 5.50
With cover, $4.25 extra.
For sixteen pages, 100 copies, without cover, . . . . 5.35
Cover, $1.75 extra.
500 copies without cover, . . . . . . . . . . . . 9.50
Cover, $4.25 extra.
For thirty-two pages, 100 copies without cover, . . . 10.70
Cover, $1.75 extra.
500 copies without cover, . . . . . . . . . . . . . 17.50
Cover, $4.25 extra.

The above figures will give perfect work on good paper.

If a larger number of reprints be desired, special rates will be given on application to THE JOURNAL. If a paper makes less than the eight pages given, a special rate will be given for the same.

JAS. P. HARRISON & CO.,
*Publishers.*

### OUR ADVERTISERS.

HUNYADI JANOS WATER.—See new advertisement of this water on page 9.

LIQUID BREAD.—This preparation, manufactured by David Nichols, of St. Louis, is one of the most valuable preparations of Malt on the market. It is for sale in this city by L. H. Bradfield, at 26 Whitehall street.

JOHN BARRY, NEW YORK.—This gentleman has an advertisement elsewhere, in THE JOURNAL, of Clinical Thermometers, manufactured by himself. They are first-class instruments, and are perfectly reliable.

W. R. CHURCH, YORKVILLE.—Elsewhere will be found an attractive advertisement of this gentleman, manufacturer of Road Carts, Phaetons, Buggies, etc. He has opened a branch house in this city, which is managed by Mr. Frank W. Earle. Those of our readers in need of Road Carts, or anything in his line, will do well to write him for prices before buying.

CAULOCOREA.—Of this preparation, which is manufactured by Dr. J. W. Lowell, Portland, Maine, Dr. S. M. Smith, of Lima, Ohio, says: "In a case of protracted metrorrhagia, accompanied with anæmia, resulting in the usual phenomina, pain in the head, with great photophobia, I prescribed caulocorea with nutritious diet, proper rest and exercise. At the end of ten days there was no recurrence of head pains. After continuing the caulocorea two months, the patient was discharged, declaring she felt stronger and livelier than for years."

HAYDEN'S VIBURNUM COMPOUND, manufactured by the New York Pharmaceutical Company, of Bedford, Mass., is a preparation that is gaining quite a reputation in this section. It is sold by Chas. O. Tyner, at 30 Marietta street. Mr. Tyner has always on hand the freshest and newest drugs and pharmaceutical preparations. Physicians can always find what they want at his store.

LUMINOUS SIGNS.—By reference to advertising page 26, our readers will find something of interest concerning these signs. They are highly endorsed by all who have them.

DR. H. G. FARR, BOSTON.—See advertisement of this gentleman, on page 18, and write him for descriptive circulars of his goods.

LACTOPEPTINE.—We know of no digestive agent more popular than Lactopeptine. If any of our readers desire to give it a trial they will be furnished with samples free by writing to the New York Pharmacal Association, New York.

